# Effectiveness of scalpel debridement for painful plantar calluses in older people: a randomized trial

**DOI:** 10.1186/1745-6215-14-243

**Published:** 2013-08-06

**Authors:** Karl B Landorf, Adam Morrow, Martin J Spink, Chelsey L Nash, Anna Novak, Julia Potter, Hylton B Menz

**Affiliations:** 1 Department of Podiatry, La Trobe University, Melbourne, Australia; 2 Lower Extremity and Gait Studies Program, La Trobe University, Melbourne, Australia; 3 School of Podiatry, University of Newcastle, Callaghan, Australia; 4 Faculty of Health Sciences, University of Southampton, Southampton, England

**Keywords:** Aged, Callosities, Foot, Mobility limitation, Pain

## Abstract

**Background:**

Plantar calluses are a common cause of foot pain, which can have a detrimental impact on the mobility and independence of older people. Scalpel debridement is often the first treatment used for this condition. Our aim was to evaluate the effectiveness of scalpel debridement of painful plantar calluses in older people.

**Methods:**

This study was a parallel-group, participant- and assessor-blinded randomized trial. Eighty participants aged 65 years and older with painful forefoot plantar calluses were recruited. Participants were randomly allocated to one of two groups: either real or sham scalpel debridement. Participants were followed for six weeks after their initial intervention appointment. The primary outcomes measured were the difference between groups in pain (measured on a 100-mm visual analogue scale) immediately post-intervention, and at one, three and six weeks post-intervention.

**Results:**

Both the real debridement and sham debridement groups experienced a reduction in pain when compared with baseline. Small, systematic between-group differences in pain scores were found at each time point (between 2 and 7 mm favoring real scalpel debridement); however, none of these were statistically significant and none reached a level that could be considered clinically worthwhile. Scalpel debridement caused no adverse events.

**Conclusions:**

The benefits of real scalpel debridement for reducing pain associated with forefoot plantar calluses in older people are small and not statistically significant compared with sham scalpel debridement. When used alone, scalpel debridement has a limited effect in the short term, although it is relatively inexpensive and causes few complications. However, these findings do not preclude the possibility of cumulative benefits over a longer time period or additive effects when combined with other interventions.

**Trial registration:**

Australian Clinical Trials Registry (ACTRN012606000176561).

## Background

Calluses are hyperkeratotic skin lesions that commonly develop on the plantar surface of the forefoot in response to mechanical stress [1]. These lesions are highly prevalent in older people, with estimates ranging from 36% to 78% in community-dwelling older populations [2-9]. Calluses are a common cause of foot pain [3], which can have a significant, detrimental impact on the mobility and independence of an older person [10]. Foot pain in older people has been associated with functional limitation, disability in activities of daily living and an increased risk of falling [3,11-15]. Therefore, appropriate management of painful corns and calluses in older people is important for maintaining functional status. Scalpel debridement is often the first treatment used for providing temporary pain relief of symptomatic calluses [16-18].

Currently, evidence from rigorous randomized trials evaluating the effectiveness of scalpel debridement for painful plantar calluses in otherwise healthy older people does not exist. To date, the clinical decision to debride these symptomatic lesions has been based largely on anecdotal evidence and non-randomized trials [19-21]. Therefore, the aim of this study was to evaluate the effectiveness of scalpel debridement of painful plantar calluses in older people.

## Methods

### Design overview

We conducted a parallel-group, participant- and assessor-blinded randomized trial. Recruitment occurred from May 2006 to November 2008. Participants were randomly allocated to receive one of two treatments: either real callus debridement, or sham callus debridement. Ethical approval for the trial was gained from the Faculty Human Ethics Committee at La Trobe University (Application number FHEC06/25). All participants provided written informed consent prior to recruitment.

### Setting and participants

Participants were recruited from patients at the La Trobe University Podiatry Clinic and from residents at the La Trobe Retirement Village, Bundoora, Australia. Participants were included in the trial if they were aged 65 years or older and had a painful plantar forefoot callus that had not been treated in the previous six weeks. Pain at the callus site needed to register at least 20 mm on a 100-mm visual analogue scale. Participants were excluded from the trial if they had any inflammatory or neurological condition that affected the feet, or if they had received foot orthoses in the previous two months, or were expecting to receive an in-shoe device during the six-week intervention period, or if they had a history of plantar forefoot ulceration in the previous three months, or if they had had a foot amputation that was proximal to the digits. Participants were also deemed ineligible if they were unable to walk household distances without an aid or were cognitively impaired (defined as a score of <7 on the Short Portable Mental Status Questionnaire [22]).

### Clinical protocol

Participants were assessed and treated at the La Trobe University Podiatry Clinic. The intervention was performed in a clinical treatment room, which was separate from the research room, where data collection was conducted. The random allocation sequence was generated in one block of 100 (50 experimental, 50 control) under the knowledge that we would recruit fewer participants than this; see section entitled ‘Sample size and statistical analysis’. The allocations were concealed from the investigators enrolling participants in sequentially numbered opaque, sealed envelopes – this system has been previously reported [23] and has been recommended by the CONSORT Statement [24] as an alternative to third-party systems. The envelope that corresponded to the participant’s study number was only opened (by the clinician providing the intervention) after the enrolled participant completed all baseline assessments and received any initial treatment (such as nail cutting). This corresponded to the time that the study intervention for the plantar forefoot needed to be allocated.

### Intervention

Following baseline assessments, which were conducted in the research room, all participants were escorted to the clinical treatment room, where they initially received treatment of nails and hyperkeratotic lesions other than those on the plantar surface of the metatarsal heads (for example, dorsal digital lesions). Once this treatment was completed, a curtain was drawn between the clinician providing the intervention and the participant, to prevent the participant from viewing the intervention for the plantar forefoot callus. From this moment, the clinician providing the intervention did not communicate with the participant. A second investigator conducting the assessment (who remained blinded to the intervention) sat next to and continued to communicate with the participant.

The ‘experimental’ intervention involved real (sharp) scalpel debridement of all plantar forefoot callus or corns on both feet. By contrast, the ‘control’ intervention involved sham debridement, where the scalpel blade was positioned upside down and the blunt edge of the blade was scraped over the surface of the callus with no removal of the lesion. We attempted to control the time taken for ‘debridement’ in the control group by spending an appropriate time for the size and thickness of the particular callus for each participant (for example, an extended time for a large, thick callus). To further mimic normal treatment, a fine paper-sanding disc (Moore’s disc) was used very lightly on the entire plantar forefoot callus in both groups. To maintain blinding while transferring participants back to the research room for data collection, a thin gauze tape (Mefix^®^) was applied over the plantar callus site and surgical booties placed over both feet, which were removed just prior to the immediate post-intervention outcome evaluation.

Following evaluation, the thin gauze tape was removed, then tincture of benzoin compound (Friar’s Balsam) and a moleskin pad was applied to both plantar forefeet, as is often done in usual care. To ensure blinding, participants were not allowed to view the plantar surface of their feet during this time. All participants (that is, both groups) received the moleskin pad and participants were advised to leave the pads on for at least two days. A follow-up appointment was arranged for outcome measurement to be repeated after six weeks.

### Outcomes and follow-up

To measure the pain at the most painful callus site, a 100-mm visual analogue scale was used. Participants were required to nominate the most painful callus site on the plantar aspect of their forefeet and they were asked to concentrate on that site when completing all outcome measures. Measurements of pain were obtained pre- and immediately post-intervention, and on a weekly basis thereafter for six weeks. Pain measurements at the initial appointment (pre- and immediately post-intervention) and follow-up appointment (six weeks post-intervention) were recorded under the supervision of the investigator conducting the assessments, who was blinded to the intervention. Five visual analogue scales were given to each participant at the conclusion of the initial appointment, for recording pain levels at home in weeks one through five post-intervention.

To measure plantar pressure, the MatScan^®^ system (Tekscan, Boston, MA), recording at a sampling rate of 40 Hz was used. Tekscan’s sensor technology has been shown to be valid and reliable for plantar pressure measurement [25,26]. The method of data collection was similar to previous studies we have conducted [25,27]. The mat was calibrated for each participant using each participant’s own bodyweight prior to testing, and a two-step gait initiation protocol was used [28-30]. Three trials were recorded [28,29], and any abnormal or aberrant footprints were discarded until three representative footprints had been recorded. As walking speed can affect plantar pressure [31], each trial was timed to ensure each participant walked at a consistent speed for each of their pressure trials. Following data collection, Research Foot^®^ software (version 5.24) was used to construct participant-specific ‘masks’ to determine peak plantar pressure (kg/cm^2^) under the most painful callus site, as specified by the participant.

To measure physical performance, a series of four balance and functional ability tests were conducted, including maximum balance range [32], a timed sit-to-stand test, an alternate step test, and a timed six metre walking test (that is, walking speed) [33]. These tests have been previously used to assess the effect of foot problems on balance and functional ability in older people [34]. All tests were performed without shoes to eliminate footwear as a variable, and maximum balance range and walking speed were normalized for height prior to analysis.

### Sample size and statistical analysis

The sample size of 80 (that is, 40 per group) was determined before beginning the trial. This sample size provided an 80% probability of detecting a clinically worthwhile difference between the interventions of 13 mm (standard deviation (SD), 20 mm) on a visual analogue scale for pain (*α* = 0.05), which was the primary outcome measure. This estimate for the sample size also factored in a 5% drop-out rate and conservatively ignored the extra precision provided by the covariate analysis we employed.

All data were analyzed by intention to treat and according to a pre-planned protocol as outlined in our clinical trial registration. For all missing data, each participant’s previous score was carried forward (that is, the last observation was carried forward). Continuous data were initially checked to ensure they did not deviate from the assumption of a normal distribution – all variables were normally distributed. To maximize precision of estimates, analysis of covariance (ANCOVA) was conducted using a linear regression approach [35]. The primary outcomes analyzed were the difference between groups in pain immediately post-intervention, and at one, three and six weeks post-intervention (making four time-points in all). All other measured variables were considered secondary outcomes.

To avoid bias in selecting covariates, we pre-specified that the baseline outcome measure would be used as the only covariate in each analysis [36]. For example, when comparing pain at each time point, adjustments were made for pain at baseline. The primary aim was to estimate the magnitude of effects, but hypothesis tests were also conducted. Mean differences, 95% confidence intervals and *P* values were calculated, and hypothesis tests were considered significant for *P* < 0.05.

An independent sample *t* test was used to determine whether there were differences between groups for time to follow-up and time spent debriding the painful plantar callus. A one-way between-group analysis of variance (ANOVA) was used to evaluate differences in walking speed between the real debridement and sham debridement groups at the three time-points that plantar pressure was measured.

## Results

Participants in this trial had a mean age of 72.5 years (SD, ±5.5); they were primarily female (63% of the sample) and presented with relatively high levels of pain at the callus site (mean 51 mm, SD, ±21), which was most often beneath the second metatarsal head. Table 1 provides the baseline characteristics of participants. Participants in the two groups had similar characteristics at baseline, although the real debridement group had slightly higher pain levels at baseline (54.6 mm vs. 47.9 mm); our ANCOVA statistical model adjusted for this.

**Table 1:** Baseline characteristics of participants

**Characteristic at baseline**	**Real debridement**	**Sham debridement**
	***n ***= **41**	***n ***= **39**
Age, years	71.8 (5.5)	73.3 (5.4)
Number of women (%)	30 (73%)	20 (51%)
Weight, kg	74.4 (13.4)	79.6 (14.8)
Height, m	1.64 (0.08)	1.66 (0.11)
Body mass index, kg/m^2^	27.9 (4.7)	29.2 (4.1)
Heel height of shoe, mm	15.1 (6.5)	14.1 (6.8)
Hallux valgus, measured using the Manchester Scale [37]	1 (0 to 3)	1 (0 to 3)
Median toe deformities (range)	1 (1 to 2)	1 (1 to 2)
Pain at callus site, mm on a visual analogue scale	54.6 (21.0)	47.9 (20.8)
Peak plantar pressure, kg/cm^2^	2.15 (0.48)	2.39 (0.42)
Maximum balance range, cm	12.0 (4.1)	11.1(2.8)
Sit-to-stand time, s	14.2 (4.6)	14.5 (4.6)
Alternate step time, s	13.7 (4.4)	13.5 (3.8)
6-m walking time, s	6.1 (1.6)	6.7 (1.6)

Values are means (SD), unless stated otherwise.

**Figure 1:**
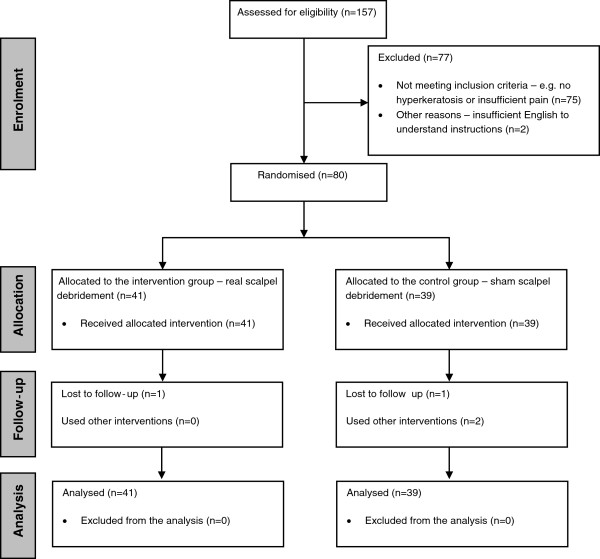
Participant flow diagram.

The progression of participants through the trial is presented in Figure 1. Two participants (one participant in each group) were lost to follow-up over the six-week period. One participant also failed to return visual analogue scales for weeks one to five post-intervention. Accordingly, we had very little missing data that required imputation as per the intention-to-treat protocol that we observed.

There was no statistically significant difference between the groups in the time to follow-up (*t*_78_ = 0.237, *P* = 0.813). However, there was a statistically significant difference between the groups in the time taken to debride (normal *versus* sham debridement) the entire plantar forefoot callus of both feet (*t*_78_ = 2.439, *P* = 0.017). The mean time for normal debridement of the plantar callus in the experimental group was 13.9 minutes (SD ±5.5) compared with the mean time of 11.0 minutes (SD ±5.1) for sham debridement in the control group.

### Primary outcomes

**Figure 2:**
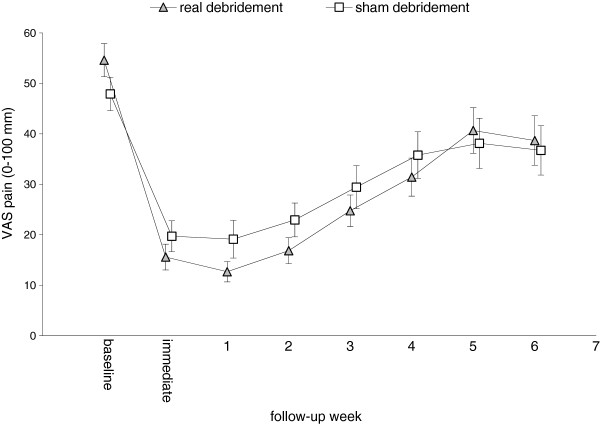
**Comparison of pain scores over time between the real debridement group and the sham debridement group.** Values are means (SD).

Compared with baseline, both groups experienced improvements in pain immediately post-intervention, and at one and three weeks post-intervention (Table 2). When comparing raw pain levels following intervention, the real debridement group had lower mean pain scores than the sham debridement group, except at weeks five and six post-intervention, where pain scores were essentially the same (Figure 2). Evaluation of Figure 2 indicates a small, systematic difference in pain scores between the two groups from immediately post-debridement through weeks one to four. However, there were no statistically significant differences at any time point (Table 2). The largest mean difference between the groups, at one week post-intervention, was -7.2 mm (favoring the real debridement group).

**Table 2:** **Mean** (**SD**) **outcome scores and ANCOVA**-**adjusted estimates of mean** (**95**% **confidence interval**) **differences between groups**

**Outcome**	**Outcome score**		**ANCOVA**-**adjusted estimates of the effects**		
	**Real debridement**	**Sham debridement**	**Difference between groups**	***t *statistic**	***P***
	***(n ***= **41)**	***(n ***= **39)**	**(95% confidence interval)**^**a**^		
**Primary outcome**:					
Reduction in pain (mm on a visual analogue scale)					
Baseline	54.6 (21.0)	47.3 (20.8)			
Immediately post-intervention	16.0 (16.4)	19.7 (19.1)	-6.0 (-13.6 to 1.7)	-1.555	0.124
1-week post-intervention	12.7 (12.9)	19.1 (23.4)	-7.2 (-15.6 to 1.3)	-1.685	0.096
3-weeks post-intervention	24.8 (20.0)	29.4 (26.7)	-6.3 (-16.7 to 4.1)	-1.213	0.229
6-weeks post-intervention	38.7 (31.4)	36.7 (30.6)	-2.2 (-15.0 to 10.5)	-0.347	0.730
**Secondary outcomes**:					
Reduction in pain (mm on a visual analogue scale)					
Baseline	54.6 (21.0)	47.3 (20.8)			
2-weeks post-intervention	16.9 (16.4)	23.0 (21.0)	-7.0 (-15.4 to 1.4)	-1.651	0.103
4-weeks post-intervention	31.4 (24.1)	35.8 (29.0)	-6.8 (-18.3 to 4.8)	-1.166	0.247
5-weeks post-intervention	40.7 (28.9)	38.1 (31.1)	-0.4 (-13.3 to 12.6)	-0.059	0.953
Peak plantar pressure (kg/cm^2^)					
Baseline	2.2 (0.5)	2.4 (0.4)			
Immediately post-intervention	2.2 (0.5)	2.4 (0.4)	0.0 (-0.1 to 0.0)	-1.091	0.279
6-weeks post-intervention	2.2 (0.5)	2.4 (0.4)	0.0 (-0.1 to 0.1)	0.106	0.916
Physical performance:					
Maximum balance range (cm)					
Baseline	12.0 (4.1)	11.1 (2.8)			
Immediately post-intervention	12.3 (3.4)	11.5 (3.6)	0.0 (-0.8 to 0.9)	0.082	0.935
6-weeks post-intervention	12.5 (3.4)	11.9 (2.8)	0.0 (-0.9 to 0.9)	-0.095	0.924
Timed sit-to-stand test (s)					
Baseline	14.2 (4.6)	14.5 (4.6)			
Immediately post-intervention	13.1 (3.7)	12.9 (3.5)	0.3 (-0.6 to 1.3)	0.725	0.470
6-weeks post-intervention	12.7 (3.0)	12.6 (3.5)	0.2 (-0.8 to 1.3)	0.475	0.636
Timed alternate step test (s)					
Baseline	13.7 (4.4)	13.5 (3.8)			
Immediately post-intervention	12.4 (2.9)	12.4 (2.9)	-0.2 (-0.9 to 0.5)	-0.659	0.512
6-weeks post-intervention	12.4 (3.1)	12.4 (3.4)	-0.1 (-1.1 to 0.8)	-0.248	0.805
6-m walking test (s)					
Baseline	6.1 (1.6)	6.7 (1.6)			
Immediately post-intervention	5.7 (1.3)	6.1 (1.3)	-0.1 (-0.3 to 0.1)	-0.687	0.494
6-weeks post-intervention	5.8 (1.3)	6.3 (1.6)	-0.1 (-0.5 to 0.2)	-0.821	0.414

^a^A negative result favors the experimental group and a positive result favors the control group.

### Secondary outcomes

Like the primary pain outcomes, the mean pain scores for both groups at two, four and five weeks post-intervention were less than the baseline scores (Figure 2). The real debridement group had lower mean pain scores than the sham debridement group at two and four weeks post-intervention. However, the ANCOVA-adjusted between-group differences in the mean pain scores for each of the secondary pain outcomes were also not statistically significant (Table 2).

Because we thought that any potential pain reductions could be associated with reductions in plantar pressure, as a result of the physical removal of the callus, we also assessed whether there were differences in plantar pressure between the two groups following the intervention. As plantar pressure is affected by walking speed, we first needed to assess whether walking speed differed between the two groups at the three time-points. There were no differences in walking speed between the two groups at pre-intervention (*F*_1-78_ = 2.115, *P* = 0.150), immediately post-intervention (*F*_1-78_ = 2.597, *P* = 0.111), and at six weeks post-intervention (*F*_1-78_ = 2.815, *P* = 0.097). Once it was ascertained that there was no difference in walking speed, we then compared the mean difference in peak pressure under the callus site between the two groups. There were no significant differences in peak pressure immediately post-intervention and at six weeks post-intervention (Table 2).

In addition, we measured physical performance, as we considered this might have been positively affected by reducing pain associated with the plantar forefoot callus. There were no significant differences in any of the balance or functional tests we performed (Table 2).

### Adverse events

There were no adverse events in the real debridement group, while two participants in the sham debridement group experienced pain levels that led them to break the trial protocol. One participant received treatment from a podiatrist outside of the trial at four weeks post-intervention and the other self-treated with a callus rasp five weeks after the intervention was administered. Both participants returned for the six-week follow-up appointment and final outcomes were measured.

## Discussion

We found that, while both groups improved, there was no statistically significant difference between real scalpel debridement and sham scalpel debridement for reducing the pain associated with forefoot plantar calluses in older people. The technique used for the sham (control) intervention has been used before [38] and involved the application of the blunt edge of a scalpel blade to all of the forefoot plantar calluses. This technique simulated real scalpel debridement without physically removing any tissue. Therefore, the reduction in pain experienced by the sham debridement group was most likely due to non-intervention effects, such as the Hawthorne effect. This is an important point, and may explain, in part, the seemingly large improvements experienced by patients receiving this intervention in clinical practice.

Although none of the ANCOVA-adjusted between-group differences were statistically significant, the unadjusted mean pain scores for both groups over the six-week period (see Figure 2) highlight that immediately post-intervention and for weeks one to four, the real debridement group systematically had less pain than the sham debridement group. The ANCOVA-adjusted difference in pain was of the order of 6 to 7 mm on a 100-mm visual analogue scale. In our original sample size calculation, we selected a value of 13 mm on a visual analogue scale for pain as a clinically worthwhile value to be able to detect, based on a range from the emergency medicine literature of 9 to 13 mm [39-41]. There are no specific reports of minimal important difference values for the visual analogue scale for painful calluses in older people. Nonetheless, we have calculated minimal important differences for plantar heel pain, which we have estimated to be of the order of 8 to 9 mm on a visual analogue scale [42]. With these values in mind, the effect of real scalpel debridement alone on pain is smaller than that considered clinically worthwhile.

Comparison of the results of this trial with previous research is difficult because the only other randomized trial comparing normal scalpel debridement with sham scalpel debridement of painful plantar calluses, by Davys and colleagues [38], was conducted in people with rheumatoid arthritis. In this trial, both groups reported a minimal reduction in pain (that is 3 mm on a 100-mm visual analogue scale) immediately post-intervention; the between-group difference was not statistically significant. However, these results cannot be generalized to people without rheumatoid arthritis as specific disease characteristics (for example, synovitis of the metatarsophalangeal joints) might influence the effectiveness of callus debridement.

Nonetheless, two non-randomized trials have evaluated the effectiveness of scalpel debridement of plantar calluses for reducing pain in people without inflammatory arthritis [19,21]. One case series, consisting of 79 participants, reported a statistically significant median difference of 59.5 mm (on a 100-mm visual analogue scale), an 86% reduction, between pre- and immediately post-intervention pain scores (*P* < 0.001) [19]. This trial included both younger and older people (ranging from 21 to 90 years). Another study, conducted on 19 older people (aged between 65 and 84 years), found a statistically significant mean pain reduction of 68% (or 25 mm) immediately after scalpel debridement (*P* < 0.001) [21]. In our trial, the mean pain improvement immediately post-intervention in the real debridement group reflects a 68% (or 38 mm) reduction on a 100 mm visual analogue scale. However, the sham debridement group also experienced a 55% (or 26 mm) pain reduction. Therefore, the overall pain reduction observed for scalpel debridement in our trial is consistent with the previous non-randomized trials, but a substantial amount of the reduction could have been due to confounding non-intervention effects, which are not accounted for in non-randomized trials. Inclusion of a control group to compare the intervention against is clearly important in determining the true effect of an intervention.

We also found that real scalpel debridement had no significant influence on plantar pressure under the forefoot or on physical performance. Changes in plantar pressure have been measured previously in a study of 15 participants (7 men and 8 women, mean age 67) with plantar callus [43]. As in our trial, it was found that debridement did not significantly alter peak plantar pressure. With regard to function, a previous case series found that callus debridement did have a beneficial significant effect on functional ability [21]. However, because this study did not include a comparison group, much of the effect observed could have been due to non-intervention effects.

Few adverse events were observed in our trial. No adverse events were experienced in the real debridement group, while two participants in the sham debridement group experienced pain levels that led them to break the trial protocol. Accordingly, scalpel debridement administered under appropriate conditions for painful plantar calluses in older people leads to minimal adverse effects. Furthermore, scalpel debridement is a relatively inexpensive intervention. So, it can be concluded that scalpel debridement of painful plantar calluses in older people is inexpensive and causes few complications, but only provides small reductions in pain when used on its own.

This trial needs to be considered in light of four limitations. First, the mean time taken to debride the plantar forefoot calluses in the experimental group was almost three minutes longer than in the control group (13.9 minutes versus 11.0 minutes). However, it can be argued that this difference did not have an undue effect, as the difference is relatively small. Second, the investigators that provided the treatment to the participants were podiatry students without long-term clinical experience. A more experienced podiatrist might have potentially debrided the callus further (during real debridement), which could have influenced the symptoms experienced by participants in the experimental group. However, the students had between 3 and 4 years of callus debridement experience, constituting between 800 and 1000 hours of clinical experience. Moreover, the change in pain in our trial was similar to previous trials that used experienced clinicians, indicating that clinical experience did not affect the generalizability of our findings [19,21]. Third, scalpel debridement was evaluated in isolation over a relatively short period (6 weeks), so our findings do not preclude the possibility of additive effects when combined with other interventions (such as foot orthoses) or cumulative benefits over a longer period. Finally, our intervention period was only 6 weeks, which is at the lower end of the range for time between debridement for calluses in clinical practice. In Australia, common return periods for callus debridement range between 6 and 8 weeks, although in the National Health Service in the United Kingdom, this can be longer [44]. From our results, it is unknown what effect a longer period of time between treatments would have on the outcomes that we measured. Consequently, the generalizability of our results, where we measured the effect over a 6-week period, might be limited when compared to longer periods between treatments.

Our trial also has a number of strengths. The results can be generalized to the wider population of community-dwelling older people seeking treatment for painful plantar calluses, and we used a common treatment method that reflected standard clinical practice. Moreover, the internal validity of our trial is assured by the rigorous methodological procedures used to obtain the results, such as allocation concealment, a sham debridement control group, participant and assessor blinding, minimal missing data, and intention-to-treat analysis.

## Conclusions

Our trial found that the benefits of real scalpel debridement for reducing pain associated with forefoot plantar calluses in older people are small and not statistically significant compared with sham scalpel debridement. When used alone, scalpel debridement is not clinically worthwhile in the short term. However, these findings do not preclude the possibility of cumulative benefits over a longer time period or additive effects when combined with other interventions.

## Abbreviations

ANCOVA: Analysis of covariance; ANOVA: Analysis of variance; SD: Standard deviation.

## Competing interests

The authors declare that they have no competing interests.

## Authors’ contributions

KBL had full access to the data and takes responsibility for the integrity of the data and the accuracy of its analysis. He also helped conceive the study, obtained funding, supervised research students and clinical staff, and analyzed and interpreted the data. AM supervised clinical staff. AM, MJS, CLN and AN acquired the data. AM, MJS, CLN, AN and JP assisted in interpreting the data. HBM conceived the study, obtained funding, supervised the research students and assisted in interpreting the data. All authors, except AM, developed the design of the study. All authors helped draft and revise the manuscript, and read and approved the final manuscript.
